# Cooling effect of fungal stromata in the *Dactylis-Epichloë-Botanophila* symbiosis

**DOI:** 10.1080/19420889.2021.1938824

**Published:** 2021-06-27

**Authors:** Marlena Lembicz, Zbigniew Miszalski, Andrzej Kornaś, Katarzyna Turnau

**Affiliations:** a Department of Plant Taxonomy, A. Mickiewicz University, Poznań, Poland; b Wladysłąw Szafer Institute of Botany, Polish Academy of Sciences, Kraków, Poland; c Institute of Biology, Pedagogical University, Kraków, Poland; d Institute of Environmental Sciences, Jagiellonian University, Kraków, Poland

**Keywords:** Ascomycota, *Botanophila*, larvae, mycelium, infrared thermography, β-carboxylation, PEPC

## Abstract

The stromata of *Epichloë* fungi are structures covering part of the stem of grasses. Under the fungal layer, still green tissues of the plant survive, although the development of the new leaves is inhibited. Stromata are the places where conidia and ascospores develop. Also, here *Botanophila* flies dine on mycelium, lay the eggs, defecate, and the larvae develop. The interaction of the three symbionts was analyzed concerning the organisms’ adaptation to understand the differences in physiology and ecology of this microenvironment that support stable symbiosis spreading presently in Europe since the beginning of the XXI century. For analysis of the infrared radiation emitted by stromata, a high-resolution infrared camera FLIR E50 was used. The visualization of stromata temperature profiles was shown in the form of pseudo-colored (false) infrared images. The ^13^C discrimination was used to characterize photosynthesis of the plant tissue enclosed within the stromata. The stromata had a substantially lower temperature than the green plant tissues. The difference reached ~5.6°C during midday hours, whereas it was smaller in the evening, reaching only ~3.6°C. The mycelium of *Epichloë* cultivated on agar showed about 2°C lower temperature in comparison to the surrounding. The plant tissues enclosed within the stroma were photosynthetically active, although this activity was of phosphoenolpyruvate carboxylase (PEPC) type and less involved in heat dissipation during the day. The stromata, built by fungal hyphae, on which fungal reproductive structures develop, form a cool shelter. This shelter provides a place for the larvae of *Botanophila* flies.

## 1. Introduction

Members of *Epichloë* (Clavicipitaceae, Ascomycota) form interactions with many grasses [1]. However, the outcome of the symbiosis in which both partners participate depends on the genotypes of these partners, the plant life stage and the environmental conditions [2]. The two partners are continuously involved in cross-talk that is facilitated by reactive oxygen species (ROS) and by the production of a wide range of substances that influence the behavior of the interaction. The cryptic stage of the endophyte is controlled by the plant [3] until the specific moment when the balance is somehow destroyed. At that moment, the fungus changes its behavior and appears as an epiphyte ready for sexual reproduction. Stromata are being formed, initially white and later becoming yellow, when perithecia with asci and ascospores are produced. Bottle-shaped perithecia are formed as a result of fungal cross-fertilization. This is possible due to anthomyiid flies of the genus *Botanophila* [4,5] that are specifically attracted by volatiles produced by the fungus [6] and carry and spread spermatia from different fungal stromata. After fertilization, flies lay eggs on fungal stromata, which are used as a food source by the emerging larvae and adults. Stromata completely or partially prevent host grasses from flowering and thus from reproducing *via* seeds.

*Dactylis glomerata* L. (orchardgrass) is a grass that often interacts with the fungus *Epichloë typhina* (Pers.) Tul. & C. Tul. (e.g. [7,8]. In Poland, *E. typhina* stromata were discovered on shoots of orchardgrass in 2002 [9]. Plants colonized by this endophyte did not form seeds. Our previous research showed a stimulatory effect of *E. typhina* stromata on photosynthetic machinery in the host plant [10]. The stromata that appeared on infected plants were often visited by *Botanophila* flies [9]. The stromata were large enough (up to 5 cm long and 0.8 cm wide) to make observations using infrared thermal camera which was found to be a useful tool to study fungus-plant-fly interactions. The presence of choke disease is a relatively recent phenomenon in Poland connected especially with anthropogenic sites [11]. The attraction of insects is an important phenomenon that enables the fungus to complete the generative cycle which may lead to transfer of this fungus to another plant species. This increases the spectrum of infected hosts and, as a result, causes serious economic losses in agricultural ecosystems (e.g. [12,13],). Our interest in the triple symbioses of *D. glomerata, E. typhina* and the fly *Botanophila* has been stimulated by the finding that there are differences in temperature between the stromata and the other plant tissues which were detected during field studies. As the insects belong to ectotherms, and thus cannot change the temperature of their own body and the close surrounding, the role of temperature is believed to be important not only for the fungus that needs to keep water content of the stromata to be able to spread ascospores but also to the insects that need appropriate temperature for egg survival during dry conditions and to develop larvae before the grass will dry out. Thus, our hypothesis is, that stromata enclosing the plant vascular tissue and inhibiting leaf growth create an ideal niche for insect development and fungal survival that result in adaptation trait allowing for the multiple symbiosis of a mutualistic type.

## 2. Materials and methods

### 2.1. Identification of fungus in plant tissues and fly

This research was carried out in the vicinity of the Campus of the Adam Mickiewicz University in Poznań (52 27.857 N, 16 55.868 E) where long term observations of orchard grass (*D. glomerata*) were carried out since 2015. All the plants taken into the investigations have been marked in the field and the leaf samples checked concerning the presence of the fungus using the nucleotide sequences of the b-tubulin gene (*tubB*) as described by the authors’ previous work [9]. DNA isolation, amplification and sequencing were performed according to the procedure by [14]. DNA sequences were deposited in GenBank (National Center for Biotechnology Information, Bethesda, MD, USA) under accession numbers HM007554–HM007560. The larvae flies present on stromata and in the vicinity of the plants were identified according to the procedure by [15,16], based on the sequence of the mitochondrial cytochrome oxidase gene (*COII*) as reported by [17,18].

### 2.2. Microscopic observations

*D. glomerata* plants infected and non-infected by *E. typhina* were deposited in pots, transported to the laboratory, sampled for the presence of the endophyte in leaves and subjected to infrared camera analysis. Sections through the stromata were hand made with the razor and observed under a fluorescent microscope NICON ECLIPSE 800. The mycelium of *E. typhina* isolated from the stromata was cultivated on PDA (Potato Dextrose Agar) in darkness at the temperature 25°C and a week old culture was used for the temperature analysis as described above.

### 2.3. Infrared thermography

The plants with a lump of soil were transplanted to 40 cm diameter pots and transported to the laboratory. Infrared studies were carried out on shoots cut off directly before the analysis in a dark chamber. Some of the tests performed several times a day were carried out on plants kept in pots outside the building at an air temperature of 20–25°C in mid-June. In this case pots were transported into the room with shaded windows. The differences were also visible under field conditions but the results were of better quality from the laboratory. For the analysis of the infrared radiation emitted by stromata, a high-resolution (240–180 pixels) infrared camera FLIR E50 with a spectral range of 7.5–13 µm and a sensitivity of 0.07°C was used. The visualization of stroma temperature profiles was shown in the form of “pseudo-coloured” (false) infrared images. Image analysis was performed using the manufacturer’s instructions. The temperature measurements of shoots with stromata, both with and without *Botanophila* larvae, were conducted in mid-June. The shoots without stromata (found within the same tuft) and those that were checked for absence of endophytes, served as controls. Stromata that were already partly eaten by the insects were not included for the analysis. Infrared pictures were also obtained from the mycelium grown on the PDA medium within Petri dishes.

### 2.4. Carbon isotope analysis in organic samples

The samples were oven dried for 24 h at 105°C before being ground to a fine powder for isotopic analysis. Isotope ratio measurements of δ^13^C were performed on a Finnigan MAT 253 Mass Spectrometer coupled with a Flash HT Elemental Analyzer in continuous flow mode. Samples were weighed in tin capsules and introduced into the combustion furnace with a temperature of 1020°C. A small volume of oxygen was added to the system to ensure the full combustion of organic compounds and their conversion into elemental gases. CO_2_ was then separated in a chromatographic column (heated to 45°C) and transferred in a carrier gas (He) via a ConFlo IV Interface to the isotope ratio mass spectrometer. International isotope standards were used to calculate the results: USGS 40, USGS 41, IAEA 600 [19].

### 2.5. Statistical analysis

Statistical comparisons were performed using Statistica 12 (StatSoft) and were considered significant at P ≤ 0.05. Data normal distribution and variance homogeneity were assessed with Shapiro-Wilk’s and Levene’s tests, respectively. If necessary, data were normalized with a log10 transformation. Differences were tested by analysis of variance (ANOVA) followed by the Tukey’s post-hoc test. The IFR analysis were carried out using 10 samples of plant stromata and 5 replications of fungal mycelia grown on PDA media. Carbon isotope analysis of plant/fungal samples were based on three bulk collective samples.

## 3. Results

**Figure 1.**
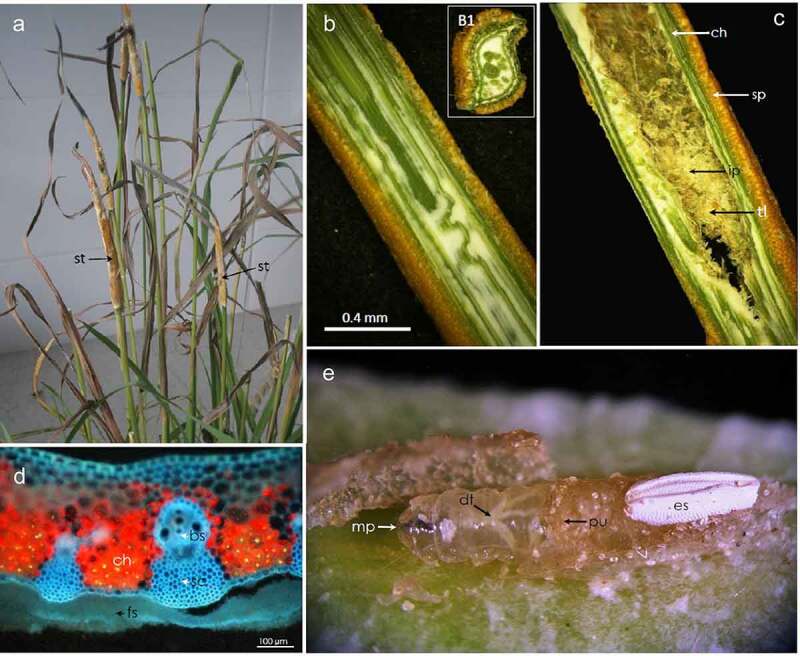
Participants in a multi-species interaction: the grass *Dactylis glomerata*, the fungus *Epichloë typhina* and a fly of *Botanophila* sp.: (a) tuft of the grass with several stromata of *E. typhina*, the fungus causing ‘choke disease’; (b) longitudinal and transversal (B1) section through the stromata; (c) section through the inside of stromata with destroyed inside of the plant stem by insects: sp – perithecial stromata, ch – chlorenchyma, ip – internal part; (d) section of stroma visualized using fluorescence microscopy, showing red autofluorescence of active chloroplasts, bright blue fluorescence of vascular tissues and dim blue fluorescence of fungal surface mycelium forming stroma: ch – chlorenchyma, sc – sclerenchyma, bs – vascular bundle, fs – fungal stromata; (e) larva of the *Botanophila* fly emerging from the chamber, with visible egg shell: mp – mouth parts, dt – digestive tract, pu – pupa, es – egg shell

**Figure 2.**
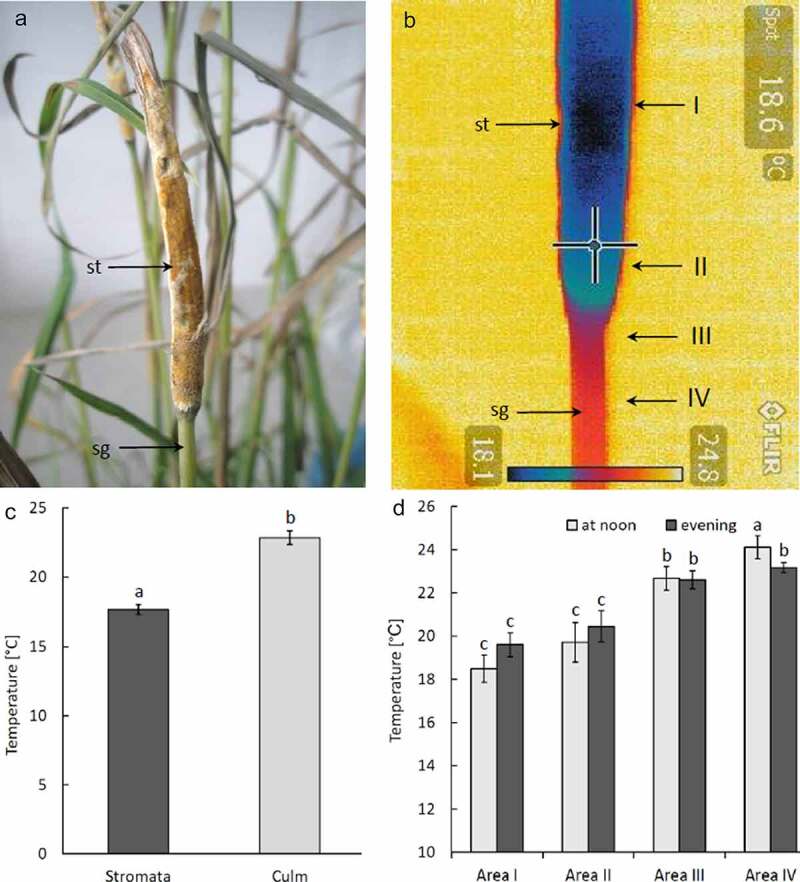
Temperature distribution in the grass culm of *Dactylis glomerata*, the fungus *Epichloë typhina* image of the stromata: (a) view of the stromata without thermovision; (b) thermovision image of the stromata developed on grass culm: st – stromata, sg – stalk grass; (c) temperature differences between inside of the stromata and grass culm below stromata measured with IR camera inside dark chamber; (d) temperature differences between individual areas (I–IV as shown in fig. 2B) at noon and in the evening measured in shaded place

**Figure 3.**
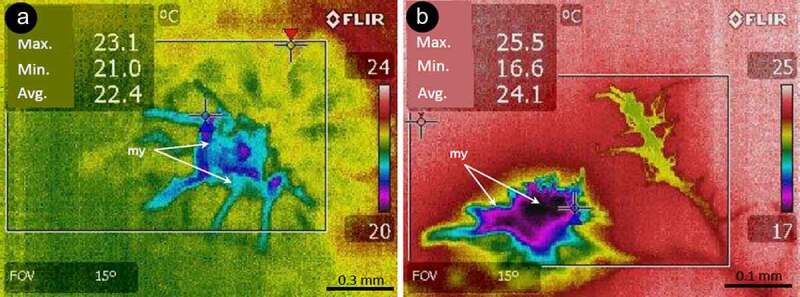
Two examples (a) and (b) of thermovision images of *Epichloë typhina* mycelium grown on PDA agar under laboratory conditions showing differences between temperature of the mycelium and agar; my – mycelium

*E. typhina* stromata often developed on still leaved stems (Figure 1(a)). At the time of the experiment the presence of stromata were visible on most plants colonized by the fungus. The mycelium overgrew them and, as a result of physical limitation of the growth, the leaves inside the stromata became characteristically bent (Figure 1(b), B1). The leaves inside stromata were still green which was visible on the cross and longitudinal sections and, despite being covered by the mycelium, they were still photosynthetically active, especially within the vascular tissues (red fluorescence of chloroplasts visible in a fluorescence microscope – Figure 1(d)). Growth inhibited leaves under the stromata were layered with the mycelium (Figure 1(b)). Numerous stromata were colonized by larvae (Figure 1(e)). The fly larvae have often been found forming corridors within the interior of the stromata and plant tissues located within them and at the later stage almost completely destroyed the internal structure, leaving dry tissue remains covered by stromata wall (Figure 1(c)). The infrared camera displayed a “thermal profile” of shoots and stromata together. No differences were found between leaf tissues of infected and non-infected plants. The stromata had substantially lower temperature than the green plant tissues. The difference reached ~5.6°C during midday hours, whereas it was smaller in the evening, reaching only ~3.6°C (Figure 2). No significant differences were found between dry stomata and plant tissues. The mycelium of *Epichloë* cultivated on agar showed about 2°C lower temperature in comparison to the surroundings (Figure 3). The alive fungus showed the guttation over the mycelium and the deposition of exudates over the mycelium in the Petri dish.

δ^13^C discrimination values in leaves of infected plants (−29.53‰ ± 0.87) were lower than in control non-infected plants (−27.91‰ ± 0.02) and for stems the difference is also visible (−28.06‰ ± 0.02 *versus* −27.41‰ ± 0.60), respectively.

## 4. Discussion

Recently, attention has been paid to the use of thermal imaging cameras in various fields of medicine (e.g. [20], and plant physiology (e.g. [21]. It was shown to be useful e.g. in genetic studies to distinguish stomatal behavior between mutants. The plants that have open stomata had colder leaves and this was visible as blue color of the false thermal image in comparison to red parts of the warmer leaves with closed stomata. As reviewed by [21], differences in leaf temperature resulted both from convection and transpiration [22,23] and depended not only on stomatal conductance to water vapor but also on plant characteristics, such as: ability to absorb light, air humidity and temperature, differences in stomatal aperture, abundance of stomata and water loss through the cuticle [24,25]. The differences between mutants were not bigger than 2°C. In case of *E. typhina* and the *D. glomerata* leaves, the temperature differed by up 3.5 to 5.6°C between the stroma and leaves of the plant in which the fungus was still in the cryptic phase and leaves free of the fungus. The temperature decrease was clearly related to the higher evaporation in the mycelium than in the plant tissues covered by the cuticle. It can be assumed that under the conditions of higher water availability in the morning, stronger evaporation was responsible for more efficient temperature decrease by the stromata. According to our measurements also the fungal mycelium growing on the agar plate and the agar itself had 2°C lower temperature than the agar surrounding the fungal colony. The higher evaporation of the fungus than of the agar is visible by the deposition of water drops on the inside upper cover of the Petri dish directly over the mycelium, but not over agar without the mycelium. Active guttation on the surface of the mycelium is the sign of fungal activity and may influence the difference in temperature between the stromata and the plant stem. This effect results from the differences in moisture contents and evaporation rates of these areas.

Using photosynthetic carbon isotope discrimination it is possible to distinguish tissues that use mostly phosphoenolpyruvate carboxylase (PEPC) for CO_2_ prefixation (β-carboxylation) from those which most of CO_2_ fixed with RubisCO. Non-typical photosynthetic green tissues (stems or veins) or typical heterotrophic tissues tend to be enriched (less negative discrimination factor) in δ^13^C compared with leaves [26] and in such tissues PEPC can be somehow more intensively involved in biomass building [27,28]. Differences in δ^13^C discriminations indicate more heterotrophic character of stems compared to leaves and also stimulating role of *Epichloë* infection on CO_2_ fixation with RubisCO (ribulose-1,5-bisphosphate carboxylase/oxygenase) visible in leaves and stems as well. Enzymatic machinery of β-carboxylating tissues can work in photosynthetic-limiting low light conditions [29]; this can be true for our experimental model *Dactylis-Epichloë* for stems and stromata tissues. Clear differences in temperature between leaves and stromata may result from: intensification of photosynthetic apparatus in plant infected with *Epichloë*, as it was also shown in our previous work [10], and low light conditions and intensification of β-carboxylating process within stromata. Low temperature of stromata can indicate also effective use of absorbed light energy resulting in lowering of light energy emission (also low temperature visible during evening hours at lower light), and also higher evaporation from increased internal area of the tissues within stromata. In addition to this, one can expect lack of HR-like response (hypersensitive response) usually responsible for increasing temperature [30] in successfully interacting *Dactylis- Epichloë* symbionts that was described in the present paper.

It still remains disputable whether the observed phenomenon affects the insects like *Botanophila*. In the populations of *D. glomerata* two *Botanophila* species were noted – *B. phrenione* and *B. dissecta* [18]. It has been often observed that the fly larvae formed corridors within the stromata interior and in the plant tissues located within them, especially when the air temperature increased, suggesting an additional benefit for the fly, the third partner in this interaction. On the other hand, it is well known that the volatile fragrances of fungal stromata are specific attractants to flies, effective at large distances [15,31]. The cooling phenomenon might be useful to decrease the emission of volatiles into air and might be the result of specific adaptation that lowers release of volatiles produced by a stroma. If this is the case, the cooling effect may not be another lure for the flies. This subject needs further research. Temperature has also important effect on insect egg development. Such a case was described for *Manduca sexta* eggs laid on *Datura wrightii* leaves that have lower temperature due to higher transpiration rates. The occurrence of cooling effect of fungal stromata is not limited solely to the *Dactylis-Epichloë* symbiosis. We observed a similar effect in the symbioses of two other grass species (*Puccinellia distans* and *Holcus lanatus*) with *Epichloë* fungi. *Botanophila* flies contribute to both these symbioses, as in the case of *Dactylis-Epichloë* symbiosis, although, the latter association is easier to study than the former due to formation of larger stromata. It is likely that a cooling effect of fungal stromata is a specific innovation related to the symbiosis of grasses with *Epichloë* fungi and *Botanophila* flies. The cooling effect is an additional trait contributing to phenotypic plasticity of the plant [10] and insect fitness, especially under climatic changes (drought).

## Supplementary Material

Supplemental Material
