# Supplementary material for: Cooling effect of fungal stromata in the Dactylis-Epichloë-Botanophila symbiosis
Source: Commun Integr Biol. 2021 Jun 27;14(1):151–7. doi: 10.1080/19420889.2021.1938824 (PMC8237986; doi:10.1080/19420889.2021.1938824)

Manuscript - with author details - MARKED VERSION

Click here to access/download
Supplementary Material - for review
Lembicz_2021 corrections in red.doc

Figure 1

Click here to access/download;Figure;Fig 1 13 V 2021.tiff

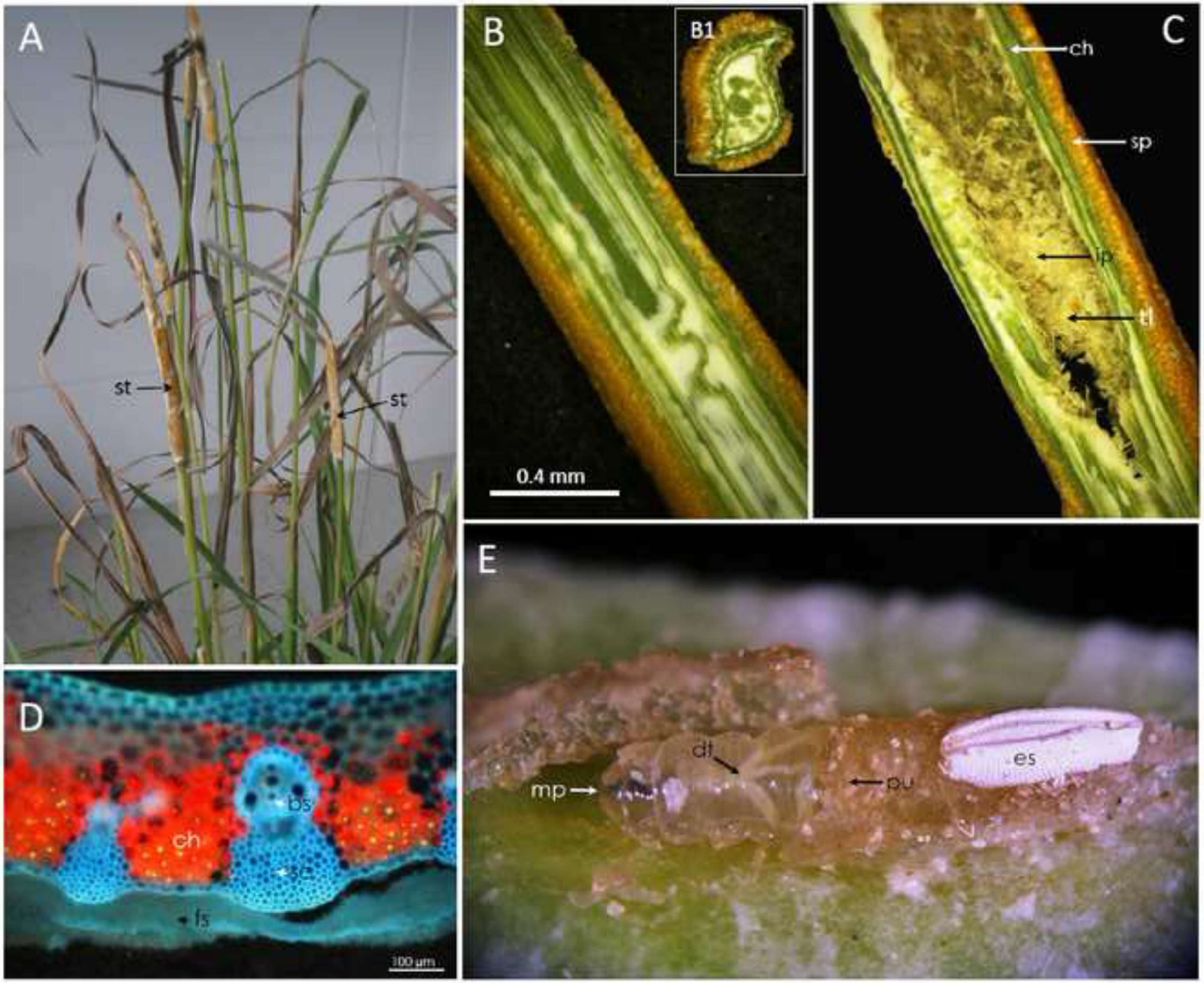

Figure 2

Click here to access/download;Figure;Fig 2 13 V 2021.tiff

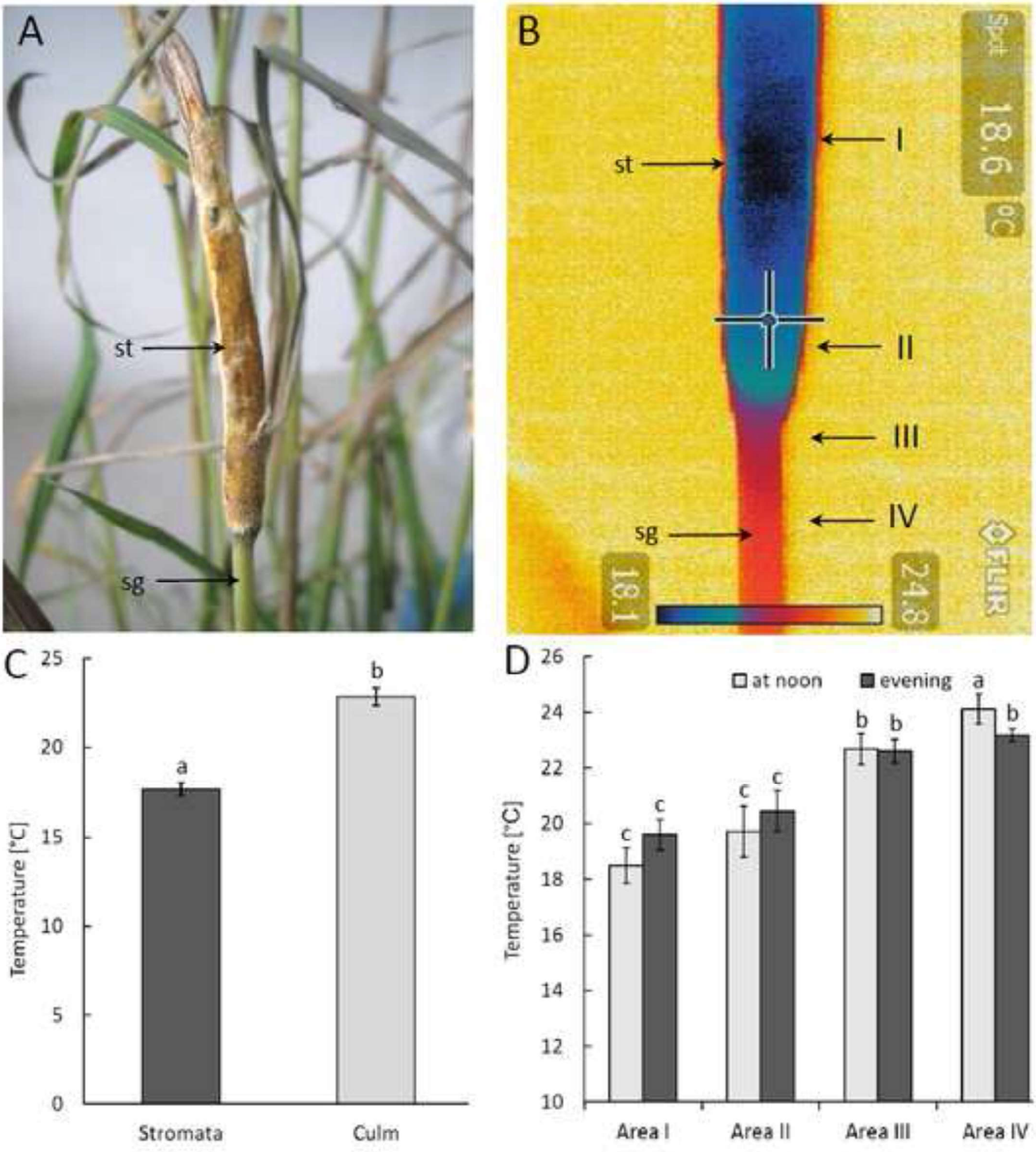

Figure 3

Click here to access/download;Figure;Fig 3 13 V 2021.tiff

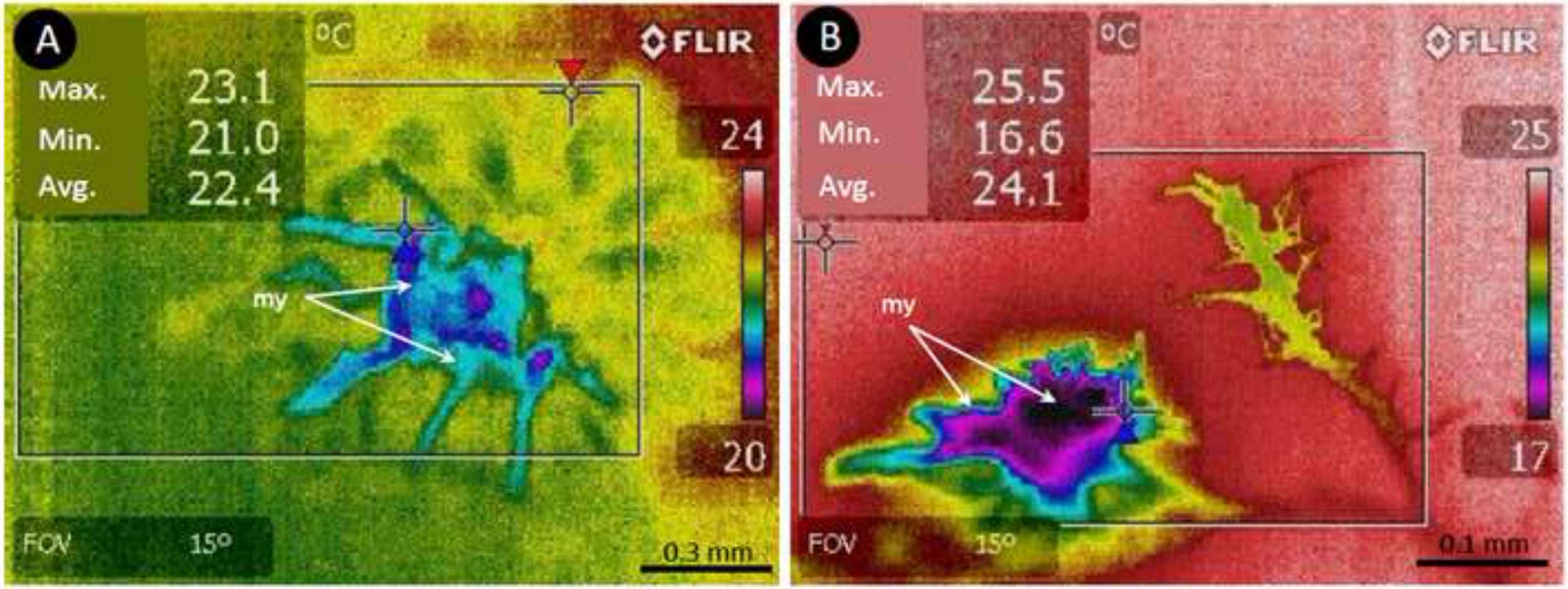

Supplement: Supplemental Material [file KCIB_A_1938824_SM9648.zip › supplementary Fig.pdf]
